# Endothelial-specific *Tie2* knockout mouse model - *Tie2*^*fl/fl*^;*Cdh5-creERT2*^*(+/−)*^: a potent tool for inducible *Tie2* deletion in vasculature across multiple organs

**DOI:** 10.1186/s12967-026-08693-y

**Published:** 2026-08-03

**Authors:** Callie Keane, Alpa Trivedi, Byron Miyazawa, Peter J. Zwiers, Grietje Molema, Shibani Pati

**Affiliations:** 1 Department of Laboratory Medicine, University of California, San Francisco, 513 Parnassus Avenue, HSE 715, San Francisco, CA 94143 USA; 2 Department of Pathology and Medical Biology, Medical Biology Section, University Medical Center Groningen, University of Groningen, Groningen, The Netherlands; 3 Department of Surgery, University of California, San Francisco, 513 Parnassus Avenue, HSE 715, San Francisco, CA 94143 USA

## Abstract

**Background:**

TEK receptor tyrosine kinase (Tie2) is predominantly expressed on endothelial cells and is known to play a critical role in vascular development and stability. Due to its role in vascular development, germline knockout of Tie2 is embryonically lethal. In this study we aimed to generate an inducible Tie2 knockout mouse model that specifically targets the mature endothelium.

**Methods:**

Mice with floxed *Tie2* exon 9 alleles (*Tie2*^*fl/fl*^) were crossed with heterozygous *Cdh5-creERT2*^*(+/−)*^ mice to generate *Tie2*^*fl/fl*^;*Cdh5-creERT2*^*(+/−)*^ (cTie2-KO) mice. Deletion of Tie2 in the endothelium of adult cTie2-KO mice was induced by intraperitoneal injection of tamoxifen, where *Tie2*^*fl/fl*^*/Cre-* (*Tie2*^*fl/fl*^) mice served as controls. Five weeks after the start of treatment, organs were evaluated for *Tie2* deletion at the DNA, RNA, and protein level. Another set of organs were collected for immunocytochemical analysis, to visually assess the expression levels of Tie2 within the tissue vasculature. The functional effect of the loss of Tie2 on the vasculature was evaluated by measuring vascular permeability in all organs in a separate set of mice.

**Results:**

*Tie2* exon 9 was deleted at the DNA level in the brains of cTie2-KO mice. All organs in cTie2-KO mice showed a significant reduction in expression of *Tie2* mRNA at multiple exon levels as compared to *Tie2*^*fl/fl*^ controls. Total protein evaluation via ELISA showed a significant reduction of Tie2 in all tissue homogenates of cTie2-KO mice. Co-localization of Tie2 with a pan-endothelial marker demonstrated that Tie2 expression was lost in the microvascular segments of all organs evaluated, as compared to control mice. Notably, vascular permeability as measured by dextran extravasation was increased in the brain parenchyma of cTie2-KO mice as compared to control mice. Deletion of Tie2 did not affect peripheral organ permeability.

**Conclusions:**

We have successfully generated a Tie2 conditional knockout mouse with significant endothelial specific deletion across multiple organs. Furthermore, at baseline we see functional deficits in cTie2-KO mice that are related specifically to blood brain barrier permeability independent of permeability in peripheral organs. This mouse model holds promise for opportunities to investigate the role of Tie2 in multiple organ vasculatures in health and disease conditions.

**Supplementary Information:**

The online version contains supplementary material available at https://doi.org/10.1186/s12967-026-08693-y.

## Background

TEK receptor tyrosine kinase (Tie2) is primarily expressed on the surface of endothelial cells. Tie2 and its ligands, angiopoietin-1 (Ang1) and angiopoietin-2 (Ang2), play a key role in angiogenesis, maintaining endothelial integrity and vascular stability [1]. Seminal papers have established that Ang1 and Ang2 interactions with Tie2 can be used to control new angiogenesis in tissues such as adipose, and sparked interest in using the Tie2 pathway to manipulate angiogenesis in health and disease [2–4]. The Tie2-angiopoeitin pathway plays a vital part in blood vessel sprouting and stabilization, such as in tip stalk formation in the retina [5]. Tie2 has been an appealing target for cancer treatment, as new drugs are developed to control angiogenesis in tumor formation and metastasis [6]. The agonist Ang1 binds to Tie2 under stable conditions, leading to autophosphorylation of the Tie2 receptor and triggers downstream phosphorylation of phosphatidylinositol 3-kinase (PI3K), Akt and ABIN. Akt activates eNOS and Survivin, facilitating cell survival and inhibiting FOXO1 translocation to the nucleus, thus preventing transcription of Ang2 [1, 7]. Furthermore, ABIN inhibits NF-kB from translocating to the nucleus, which suppresses the transcription of inflammatory genes [8]. Under destabilizing conditions, such as vascular injury or sepsis, the Tie2 antagonist Ang2 becomes more prevalent as endothelial cells release Ang2 from Weibel-Palade bodies. As a result, Ang1 mediated Tie2 phosphorylation is inhibited allowing the transcription of more Ang2 that outcompetes Ang1 in binding the receptor [9, 10]. This binding of Ang2 to Tie2 receptor results in an overall loss of cell stability and vascular compromise.

Study of the role of Tie2 in physiology and disease is complicated, as deletion of Tie2 in mouse endothelium is embryonically lethal [2]. The creation of a conditional Tie2 knockout mouse has circumvented this limitation, allowing for normal vascular development until time of evaluation. In previous inducible Tie2 knockout models, the focus has been on organ related Tie2 expression and not broadly across all organs or endothelium [11, 12]. In our previous study, a conditional Tie2 knockout mouse model, *Tie2*^*floxed/floxed*^;*end-SCL-Cre-ER*^*T*^, displayed only moderate levels of Tie2 knockout in the heart, lungs and aorta. However, the Cre recombinase expression and activity was highly tissue dependent and led to a heterogenous pattern of Tie2 deletion in individual organs as well as across organs. This limits the use of the mouse, as we were not able to observe homogeneous deletion across multiple organs for studies that may want to evaluate the role of Tie2 in disease and injury models [13].

In this study, we aim to generate a transgenic mouse model by targeting the endothelial expression of Tie2. Instead of targeting the hematopoietic stem cells, here we focused on the mature vasculature expressing VE-Cadherin. We achieved this goal by crossing *Tie2*^*fl/fl*^ mice with *Cdh5-cre-ERT2* mice. These mice express Cre-recombinase fused to the mutated human estrogen receptor (ERT2) driven by the VE-Cadherin promoter. Upon treatment with tamoxifen, the CreERT2 in the cell is induced which enables its translocation to the nucleus to excise exon 9 from the *Tie2* gene, creating a mouse with conditional knockout of Tie2 in endothelial cells [13]. We demonstrate substantial endothelial specific deletion of Tie-2 in multiple organs, and show that Tie2 plays a fundamental role in the maintenance of the integrity of the blood brain barrier. It is our future goal to utilize this model to investigate the function of Tie2 in traumatic brain injury (TBI).

## Methods

### Animal protocols

All animal procedures were performed with the approval of the UCSF Institutional Animal Care and Use Committee. The experiments were conducted in compliance with the National Institutes of Health guidelines on the use of laboratory animals. All animals were housed in a room with access to food and water, controlled temperature and a 12:12 light dark cycle.

### Generation of cTie2-KO mice

Mice with floxed *Tie2* exon 9 alleles (*Tie2*^*fl/fl*^) provided from the lab of Dr. Grietje Molema (University Medical Center Groningen, Groningen University, the Netherlands) were crossed with *Cdh5-creERT2*^*(+/−)*^ (mice purchased from Taconic, Rensselaer, NY) to generate *Tie2*^*fl/−*^;*Cdh5-creERT2*^*(+/−)*^ and *Tie2*^*fl/−*^;*Cre-* littermates. DNA was isolated from tail tissue using the QIAamp^®^ DNA Mini Kit (Qiagen, Germantown, MD, USA) according to manufacturer protocols. PCR was performed using the Amplitaq ^®^ Gold Fast PCR Master Mix (Applied Biosystems, Foster City, CA, USA) according to standard protocols using the primers 5’-GGGCTGCTACAATAGCTTTGG-3’ and 5’-GTTATGTCCAGTGTCAATCAC-3’. PCR amplified sequences were sent to Transnetyx (Cordova, TN, USA), which were used to design strain probes to genotype litters. Pups from subsequent litters were genotyped by Transnetyx at 21 days of age. *Tie2*^*fl/−*^;*Cdh5-creERT2*^*(+/−)*^ mice were then crossed with *Tie2*^*fl/fl*^ mice to generate *Tie2*^*fl/fl*^;*Cdh5-creERT2*^*(+/−)*^ and *Tie2*^*fl/fl*^ littermates.

Knockout of Tie2 in the endothelium was then induced in both male and female littermates, when the mice were 8 weeks of age. Intraperitoneal injection of tamoxifen (LGC Standards, Teddington, Middlesex, UK) was done three times per week for three weeks in *Tie2*^*fl/fl*^;*Cdh5-creERT2*^*(+/−)*^ (cTie2-KO), and *Tie2*^*fl/fl*^ control littermates at a dose of 130 mg/kg body weight. Five weeks after the start of treatment, mice were sacrificed and the lung, liver, spleen, kidney, and brain were harvested and flash frozen on dry ice or isopentane and stored at -80 °C. Another set of mice were generated to evaluate organ barrier permeability.

### DNA extraction and PCR confirmation of knockout

DNA was isolated from tissue of mice treated with tamoxifen using the QIAamp^®^ DNA Mini Kit (Qiagen) according to manufacturer protocols. PCR was performed using the Amplitaq^®^ Gold Fast PCR Master Mix (Applied Biosystems) according to standard protocols using the primers 5’-GGGCTGCTACAATAGCTTTGG-3’ and 5’-GTTATGTCCAGTGTCAATCAC-3’. PCR product was run using E-Gel™ EX 2% Agarose gels and imaged using the E-Gel™ Power Snap Electrophoresis System (Invitrogen, Waltham, MO, USA).

### RNA extraction and quantitation by qPCR

Total RNA was isolated from organs of mice treated with tamoxifen using the AllPrep^®^ DNA/RNA Mini Kit (Qiagen) according to manufacturer’s protocols. cDNA was generated using the High-Capacity cDNA Reverse Transcription Kit (Applied Biosystems). qPCR was performed for each sample using the QuantStudio 6 Flex system and predesigned TaqMan^®^ Gene Expression Assay primers (Table 1) (Thermo Fisher Scientific, Waltham, MO, USA). *Pecam-1* was normalized to the housekeeping gene, *Gapdh* and then all vasculature related markers were normalized to *Pecam-1* for the lung, liver, kidney and brain. All markers were normalized to *Gapdh* for the spleen, as *Pecam-1* showed significant changes in expression when normalized to *Gapdh*.

**Table 1:** Taqman primers used in qPCR assays of *Tie2* mRNA expression and mRNA expression of related genes

Gene Symbol	Gene Name	Assay ID
*Tek*	Endothelial-specific receptor tyrosine kinase	Mm00443242_m1
*Tek*	Endothelial-specific receptor tyrosine kinase	Mm00443243_m1
*Tek*	Endothelial-specific receptor tyrosine kinase	Mm01256894_m1
*Gapdh*	Glyceraldehyde-3-phosphate dehydrogenase	Mm99999915_g1
*Pecam1*	Platelet/endothelial cell adhesion molecule 1	Mm00476702_m1
*Tie1*	Tyrosine kinase with immunoglobulin-like and EGF-like domains 1	Mm00441786_m1
*Angpt1*	Angiopoietin 1	Mm00456503_m1
*Angpt2*	Angiopoietin 2	Mm00545822_m1
*Cdh5*	Cadherin 5	Mm00486938_m1

### Protein lysate preparation and quantitation

Tissue of mice treated with tamoxifen was homogenized in RIPA buffer and Halt™ protease and phosphatase inhibitor (Thermo Fisher Scientific), sonicated for 4 pulses at a time, 1 s each pulse at an amplitude of 70% to prepare homogenates using the Fisherbrand™ Model 120 Sonic Dismembrator (Fisher Scientific, Waltham, MO, USA). Homogenates were centrifuged at 13,000 RPM at 4 °C for 30 min, then the supernatant was collected and stored at -80 °C for use in subsequent assays. Total protein content was assayed using the Pierce™ BCA Protein Assay (Thermo Fisher Scientific). Tie2 protein content was assayed using the Mouse TIE2 ELISA kit- Intracellular (Abcam, Waltham, MA, USA) according to manufacturer instructions.

### Histological analysis of Tie2 expression by immunocytochemistry

Another set of organs were collected and flash frozen in isopentane to evaluate the expression levels of Tie2 in the vasculature. Brains were sectioned at 20 μm and lungs, kidneys, spleens, and livers were sectioned at 10 μm thickness on the Leica CM 1950 Cryostat (Leica Biosystems, Deer Park, IL, USA) and collected on SuperFrost Plus slides (Thermo Fisher Scientific). Sections were fixed in cold 95% ethanol for 20 min followed by cold 100% acetone for 1 min. Sections were then stained using antibodies against Tie2 (Abcam Limited, Cambridge, UK) and Pecam-1 (MilliporeSigma, Burlington, MA, USA). Alexa Fluor 488, 594 and 647 conjugated secondary antibodies were used for detection (Thermo Fisher Scientific). Images were captured with a Nikon Eclipse 80i microscope (Nikon Instruments Inc, Melville, NY, USA) with a SPOT RT-sCMOS camera (SPOT Imaging, Sterling Heights, MI, USA) at 20x magnification.

### Organ vascular permeability

Five weeks after the start of tamoxifen treatment, cTie2-KO and Tie2^fl/fl^ littermates were injected retro-orbitally with 200 ul of 0.2 mg/ml 10kD IR680 tagged dextran (Thermo Fisher Scientific), and allowed to circulate for 30 min. The mice were transcardially perfused with 30 ml of cold PBS, all organs were excised and the brain was sliced into 2 mm coronal sections. All organs were scanned on the Odyssey CLX Imager (LI-COR Biotechnology, Lincoln, NE, USA). Dye intensity was quantitated per organ area using the Image Studio Software (LI-COR Biotechnology). For brain slices, fluorescence intensity across all slices was summed to represent total organ permeability.

### Statistical analyses

For *Tie2* mRNA analysis we decided a priori to run a one-tailed Student’s t-test given prior verification of exon 9 deletion at the genomic level. For all other two-group comparisons, data was analyzed using the two-tailed Student’s t-test when data was normally distributed. If data was not normally distributed, the Mann-Whitney test was used. Weight comparisons were performed using a repeated measures two-way ANOVA. All statistical analyses were performed using GraphPad Prism 10 software, (Dotmatics, Boston, MA, USA). Differences between groups were considered significant with *p* ≤ 0.05.

## Results

### Generation of cTie2-KO mice

**Fig. 1:**
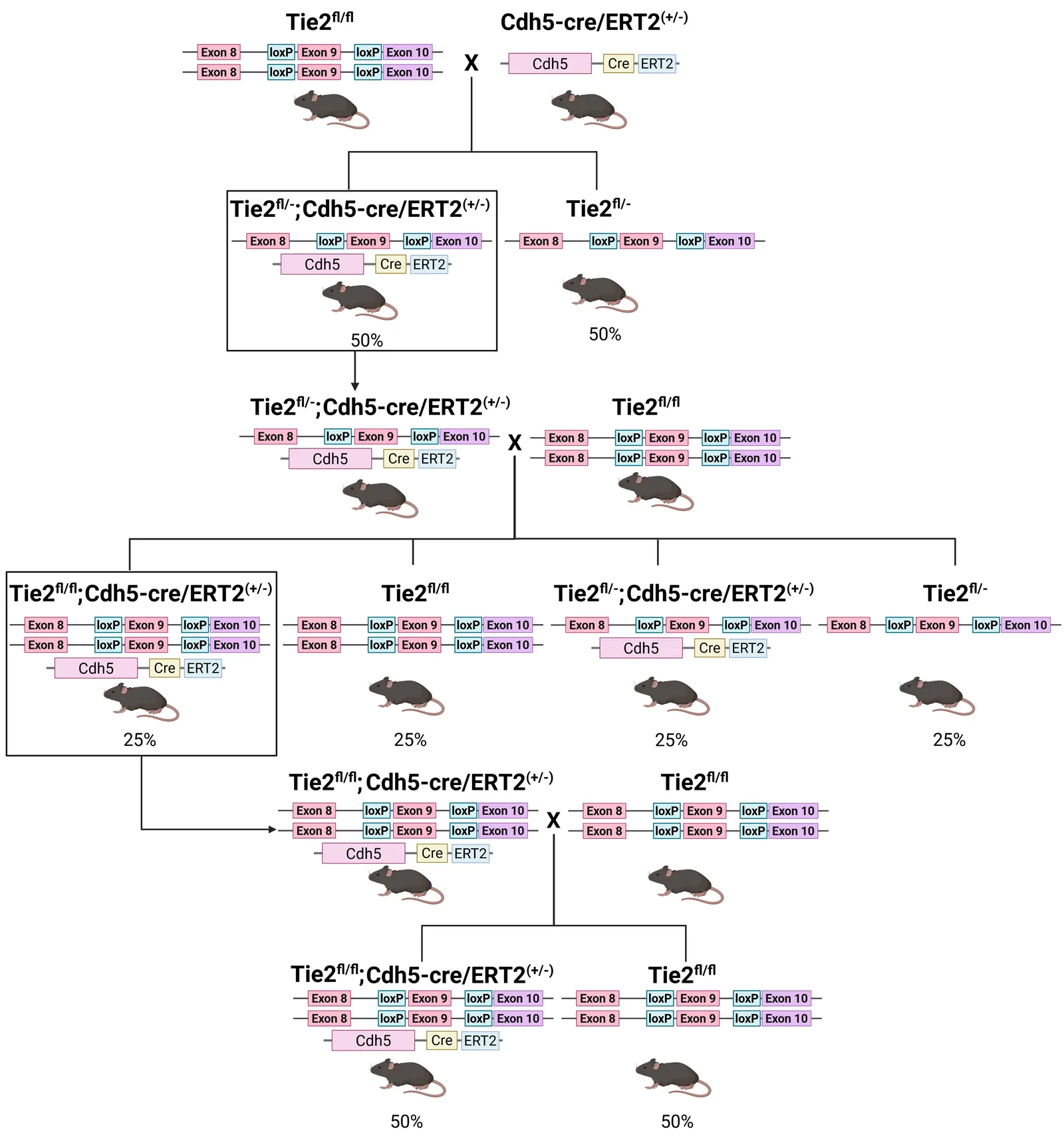
Schematic of *Tie2*^*fl/fl*^;*Cdh5-creERT2*^*(+/−)*^ breeding strategy. *Tie2*^*fl/fl*^ mice were initially crossed with *Cdh5-creERT2*^*(+/−)*^ mice to generate heterozygous *Tie2*^*fl/−*^;*Cdh5-creERT2*^*(+/−)*^ mice. These mice were then bred with *Tie2*^*fl/fl*^ mice from a separately maintained colony to generate experimental mice. For colony maintenance, *Tie2*^*fl/fl*^;*Cdh5-creERT2*^*(+/−)*^ were bred with *Tie2*^*fl/fl*^ mice from a separately maintained colony. *[Schematic created with* https://BioRender.com]

*Tie2*^*fl/fl*^ mice were crossed with *Cdh5-cre-ER*^*T2(+/−)*^ mice, both on a C57Bl/6J background. The resulting *Tie2*^*fl/−*^;*Cdh5-cre-ER*^*T2(+/−)*^ were crossed with *Tie2*^*fl/fl*^ mice to generate *Tie2*^*fl/fl*^;*Cdh5-cre-ER*^*T2(+/−)*^ mice, making up 25% of the offspring. Subsequently, we crossed *Tie2*^*fl/fl*^;*Cdh5-cre-ER*^*T2(+/−)*^ offspring with *Tie2*^*fl/fl*^ mice from an independently maintained *Tie2*^*fl/fl*^ colony to create and maintain a stable population of mice for further experiments. This results in 50% *Tie2*^*fl/fl*^ and 50% *Tie2*^*fl/fl*^;*Cdh5-cre-ER*^*T2(+/−)*^ offspring (Fig. 1). Litters were healthy, with normal sizes of 5–10 pups/litter and an even split of males and females.

**Fig. 2:**
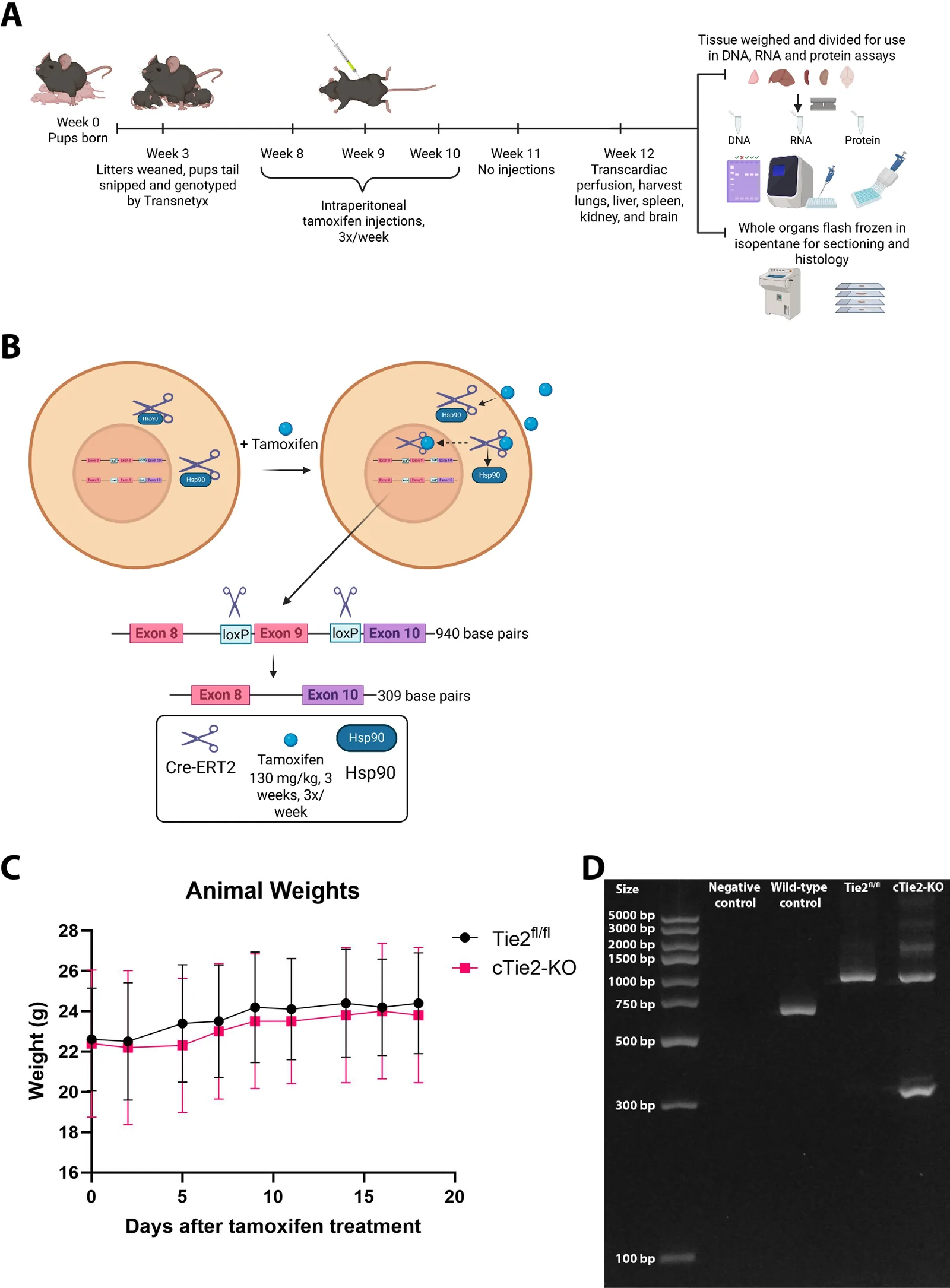
Tamoxifen injection induces knockout of *Tie2* exon 9 without causing significant health changes to cTie2-KO mice. (**A**) Diagram depicting the timeline of tamoxifen injection and organ collections. (**B**) Diagram depicting the deletion of exon 9 of the Tie2 gene by using tamoxifen to induce Cre-ERT2 and excision of the gene at the floxed sites. (**C**) Graph depicting the trend of animal weights over the three weeks of tamoxifen treatment. RM-Two-way ANOVA, graph shows mean ± SD, *n* = 10 mice per group. No significant interaction, *p* ≥ 0.999. No effect of age, *p* = 0.3809. No effect of genotype, *p* = 0.2453. (**D**) After treatment with tamoxifen, PCR was performed to depict the presence of excised exon 9 (309 base pairs) in cTie2-KO mice as compared to the presence of exon 9 at the original site without deletion (940 base pairs) in *Tie2*^*fl/fl*^ control mice. *[Diagrams created with* https://BioRender.com]

After litters reached 8 weeks of age, both males and females, were treated with tamoxifen 3 times a week for three weeks, and then their tissue was harvested at the fifth week after start of treatment for downstream analysis (Fig. 2A). Tamoxifen treatment causes heat shock protein 90 (Hsp90) to disassociate from the Cre-ERT2 protein, which then enters the nucleus and cuts the DNA at loxP sites, which in the *Tie2*^*fl/fl*^;*Cdh5-cre-ER*^*T2(+/−)*^ mice are two sites flanking *Tie2* exon 9, creating a strand of DNA missing exon 9 of the *Tie2* gene (Fig. 2B). Tamoxifen treatment had no observed effect on body weight when comparing cTie2-KO mice and *Tie2*^*fl/fl*^ littermates (Fig. 2C). Following treatment of mice with tamoxifen, in cTie2-KO mice, PCR analysis of DNA from the brain shows a band at around 309 bp and at around 940 bp, indicating deletion of exon 9. In *Tie2*^*fl/fl*^ littermate controls, PCR shows a single band at around 940 bp, showing the floxed exon 9 was not affected by tamoxifen treatment (Fig. 2D).

### Decreased *Tie2* mRNA expression in cTie2-KO mice in all organs

We next examined if Tie2 was specifically deleted in the endothelial cells by normalizing *Tie2* mRNA levels to *Pecam-1* in all organs. To verify its suitability as a reference gene, *Pecam-1* mRNA was normalized to *Gapdh* in *Tie2*^*fl/fl*^ and cTie2-KO mice. There were comparable levels of *Pecam-1* mRNA between both genotypes in all organs except the spleen. In the spleen, there was 10-100-fold less *Pecam-1* expression as compared to the other organs. Moreover, *Tie2* deletion significantly reduced the amount of *Pecam-1* expression in cTie2-KO mice as compared to Tie2^fl/fl^ mice (Supplemental Fig. 1). Hence, for follow-up analysis spleen *Tie2* mRNA was normalized to *Gapdh* whereas all other organ *Tie2* mRNA was normalized to *Pecam-1*.

**Fig. 3:**
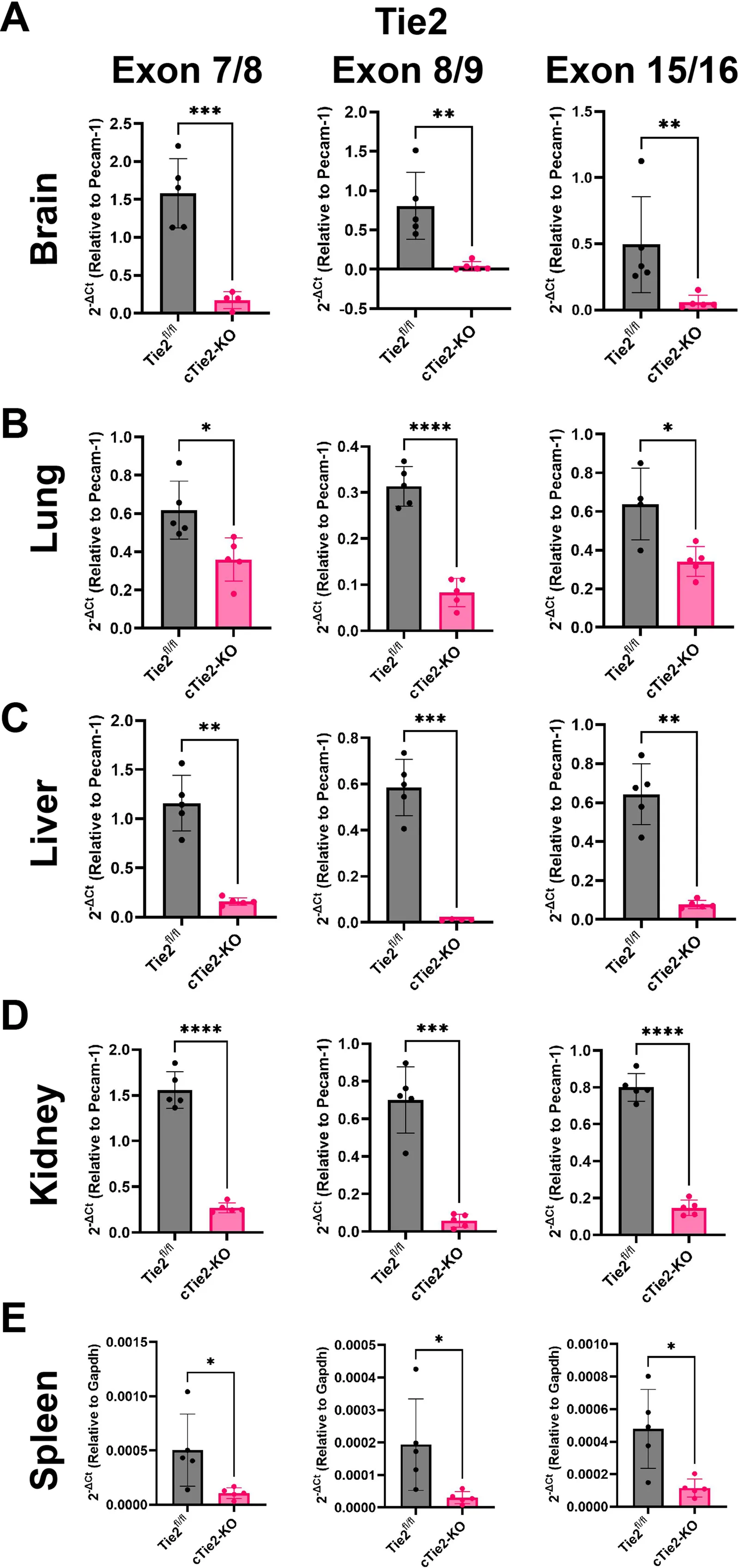
qPCR analysis of *Tie2* expression at three exon junctions, exon 7/8, exon 8/9 and exon 15/16 shows significant knockdown of *Tie2* mRNA in cTie2-KO mice. (**A**) Significant reduction of *Tie2* mRNA expression relative to *Pecam-1* in the brain in cTie2-KO mice as compared to *Tie2*^*fl/fl*^ littermates. Exon 7/8 unpaired one-tailed t-test, columns show individual values and mean ± SD, *n* = 4–5 mice per group, ***p ≤ 0.001. Exon 8/9 and exon 15/16 Mann-Whitney test, columns show individual values and mean ± SD, *n* = 5 mice per group, **p ≤ 0.01. (**B**-**D**) Significant reduction of *Tie2* mRNA expression relative to *Pecam-1* in the lung, liver, and kidney in cTie2-KO mice as compared to *Tie2*^*fl/fl*^ littermates. Unpaired one-tailed t-test, columns show individual values and mean ± SD, *n* = 4–5 mice per group. (**E**) Treatment with tamoxifen shows a significant reduction of *Tie2* mRNA expression relative to *Gapdh* in the spleen in cTie2-KO mice as compared to *Tie2*^*fl/fl*^ littermates. Unpaired one-tailed t-test, columns show individual values and mean ± SD, *n* = 5 mice per group, *p ≤ 0.05, **p ≤ 0.01, ***p ≤ 0.001, ****p ≤ 0.0001

In cTie2-KO mice, *Tie2* mRNA expression was significantly reduced in all organs assayed as compared to expression in control *Tie2*^*fl/fl*^ littermates. To confirm that there was a reduction in *Tie2* mRNA expression and investigate the possibility of truncated mRNA forms, we used three primer sets that bound to exons 7/8, 8/9 and 15/16 of the *Tie2* cDNA. The results of all three primer sets were comparable and showed a significant reduction in *Tie2* expression across all three exon sites, confirming that the reduction in *Tie2* mRNA levels in all organs examined (Fig. 3).

### Reduction in *Tie2* mRNA expression in cTie2KO mice has no effect on mRNA expression of associated vascular proteins

We performed additional qPCR on cDNA isolated from each organ to determine whether the loss of *Tie2* impacts the expression of *Tie1*, which is associated with the Tie2 receptor, or its ligands, *Angiopoietin 1*, and *Angiopoietin 2*. We also assessed the expression of *VE-Cadherin*, as a marker of the adherens junction protein of the endothelial cells. The mRNA levels of the examined vascular markers were unchanged between cTie2-KO mice and *Tie2*^*fl/fl*^ mice (Table 2). The significant reduction of *Tie2* mRNA expression did not affect the mRNA levels of associated vascular proteins.

**Table 2:** *Tie2* exon 9 deletion has no effect on mRNA expression of associated proteins in the Tie2 pathway

		*Cdh5* 2^− dCt^ ± SD	*Ang1* 2^− dCt^ ± SD	*Ang2* 2^− dCt^ ± SD	*Tie1* 2^− dCt^ ± SD
Brain	*Tie2* ^*fl/fl*^	5.30 ± 2.573	2.169 ± 1.997	0.06235 ± 0.04849	0.8024 ± 0.3343
	cTie2-KO	4.303 ± 2.355	2.518 ± 1.451	0.1639 ± 0.08650	0.4814 ± 0.2617
	p-value	0.5651	0.7690	0.0775	0.2376
Lung	*Tie2* ^*fl/fl*^	4.900 ± 1.384	0.5813 ± 0.1806	0.02128 ± 0.006223	0.7105 ± 0.4230
	cTie2-KO	4.186 ± 1.296	0.5964 ± 0.2822	0.03037 ± 0.006013	1.016 ± 0.2570
	p-value	0.4243	0.9226	0.0659	0.2121
Liver	*Tie2* ^*fl/fl*^	6.918 ± 3.213	0.1057 ± 0.03579	0.02706 ± 0.01638	1.000 ± 0.2924
	cTie2-KO	8.370 ± 2.191	0.1156 ± 0.04020	0.05030 ± 0.02404	0.7900 ± 0.1807
	p-value	0.4311	0.6909	0.1169	0.2156
Kidney	*Tie2* ^*fl/fl*^	3.370 ± 0.7815	0.7952 ± 0.2388	0.1009 ± 0.04387	1.168 ± 0.3211
	cTie2-KO	3.156 ± 0.2284	0.7809 ± 0.3188	0.1779 ± 0.06319	1.031 ± 0.1815
	p-value	0.5841	0.9382	0.0596	0.4375
Spleen	*Tie2* ^*fl/fl*^	0.006266 ± 0.002851	0.0008780 ± 0.0003070	0.0002313 ± 5.422e-005	0.004995 ± 0.001242
	cTie2-KO	0.004 ± 0.003	0.0005304 ± 8.133e-005	0.0002071 ± 8.303e-005	0.003188 ± 0.001313
	p-value	0.3847	0.0630	0.6013	0.0559

qPCR assay of expression of *Cdh5*, *Angiopoietin 1*, *Angiopoietin 2* and *Tie1* relative to *Pecam-1* for the brain, lung, liver, and kidney, and relative to *Gapdh* for the spleen. Analysis of these genes showed no significant change in mRNA levels for all organs. Unpaired two-tailed t-test, *n* = 4–5 mice per group

### Decreased Tie2 protein in all organs of cTie2-KO mice

**Fig. 4:**
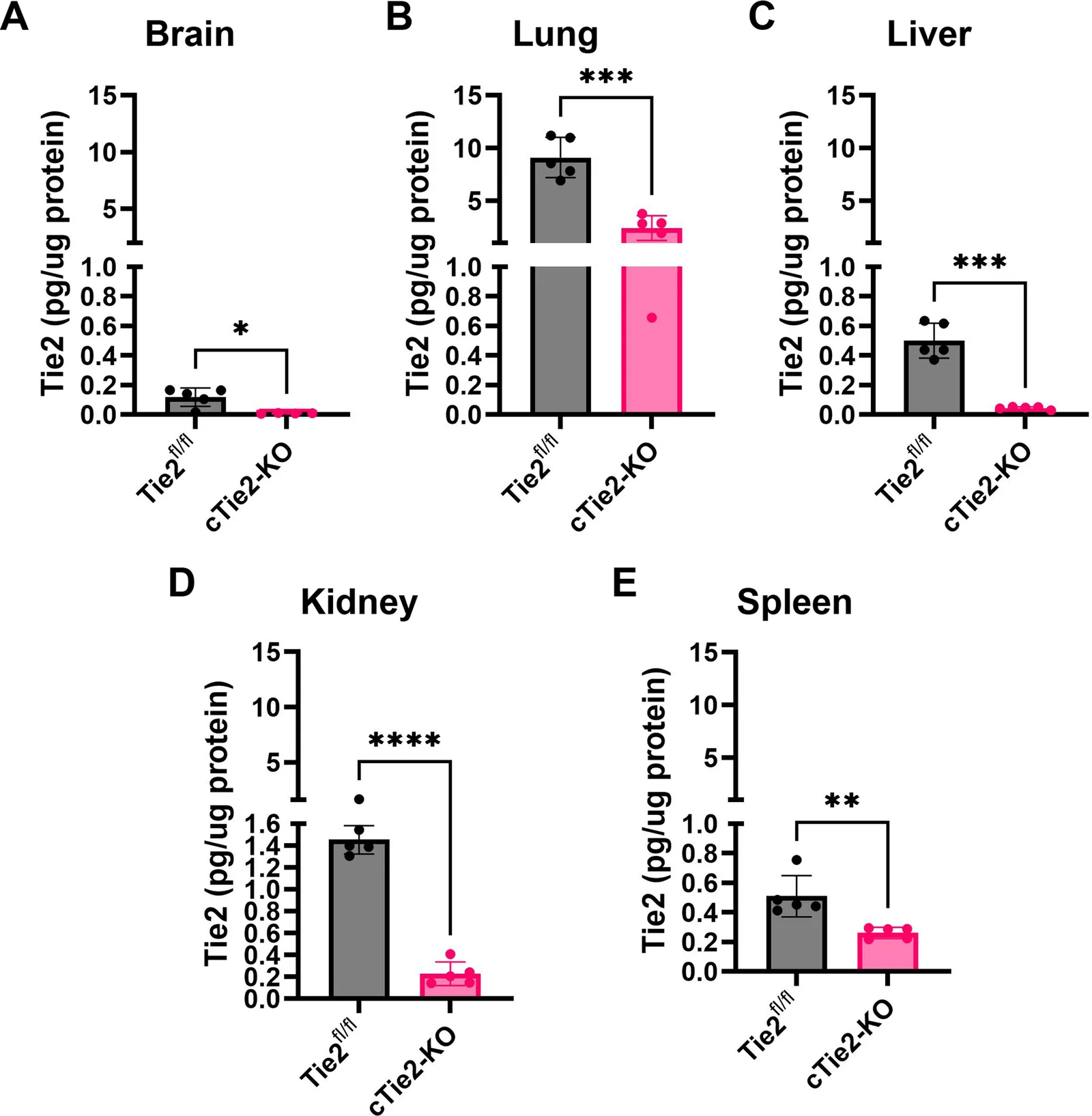
Tie2 protein in all organs is significantly reduced in cTie2-KO mice after tamoxifen treatment. (**A**-**D**) Significant reduction of Tie2 protein content in the brain, lung, liver, and kidney. Columns show individual values and mean ± SD. Unpaired two-tailed t-test, *n* = 5 mice per group, *p ≤ 0.05, ***p ≤ 0.001, ****p ≤ 0.0001. (**E**) Spleen in cTie2-KO mice as compared to *Tie2*^*fl/fl*^ littermates. Columns show individual values and mean ± SD. Mann-Whitney test, *n* = 5 mice per group, **p ≤ 0.01

We performed an ELISA on organ homogenates to quantify Tie2 protein expression. In all tissues assayed, cTie2-KO mice showed a significant reduction of Tie2 protein per microgram of total protein as compared to Tie2 protein expression in *Tie2*^*fl/fl*^ littermates (Fig. 4).

### Vascular loss of Tie2 protein in all organs in cTie2-KO mice

**Fig. 5:**
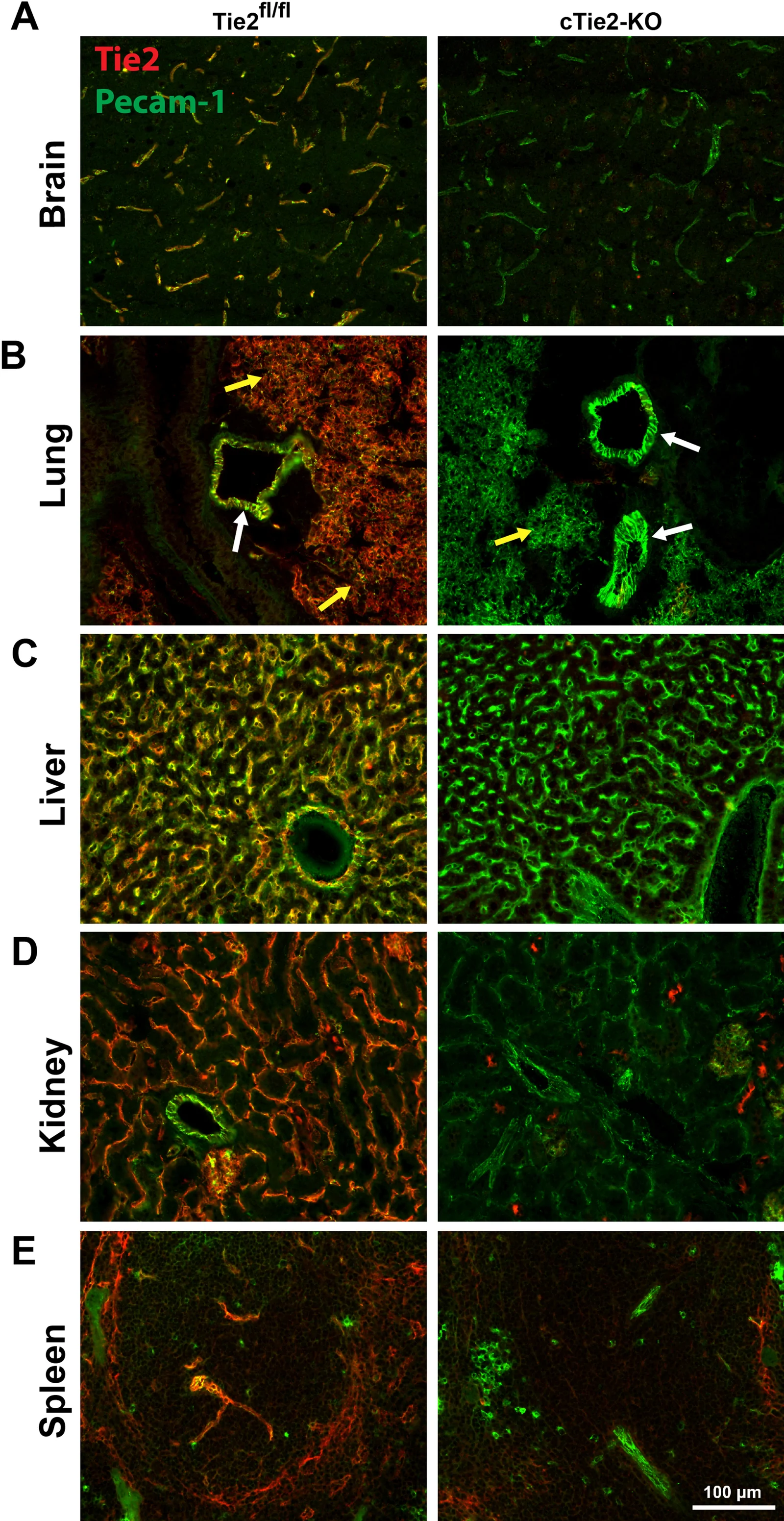
Visualization of loss of Tie2 expression in cTie2-KO mice by immunocytochemistry. *Tie2*^*fl/fl*^ sections show Tie2 expression (red) and colocalization with Pecam-1, a pan-endothelial marker (green). The cTie2-KO mouse shows a loss of Tie2 in Pecam-1 positive blood vessels in the (**A**) Brain cortex (**B**) Lung alveoli (yellow arrow) and large blood vessels (white arrow) (**C**) Liver (**D**) Kidney (**E**) Spleen. Scale bar shown in bottom right image

We next asked if the loss of Tie2 was restricted to the vascular structures in the tissue. Pecam-1 was used as a vascular marker and the co-localization of the Tie2 with the vascular marker was evaluated in the tissue sections. Lung, liver, spleen, kidney and brain sections fluorescently stained with Tie2 show visibly less Tie2 expression in cTie2-KO mice as compared to *Tie2*^*fl/fl*^ littermate controls (Supplemental Fig. 2). Additionally, the merged images of Tie2 and Pecam-1 show the loss of Tie2 is endothelial specific, as noted by the loss of overlap of the two markers in the cTie2-KO sections (Fig. 5). This staining confirms the qPCR and ELISA data and demonstrates endothelial specific loss of Tie2.

### Increased vascular leak in the brain in cTie2-KO mice

**Fig. 6:**
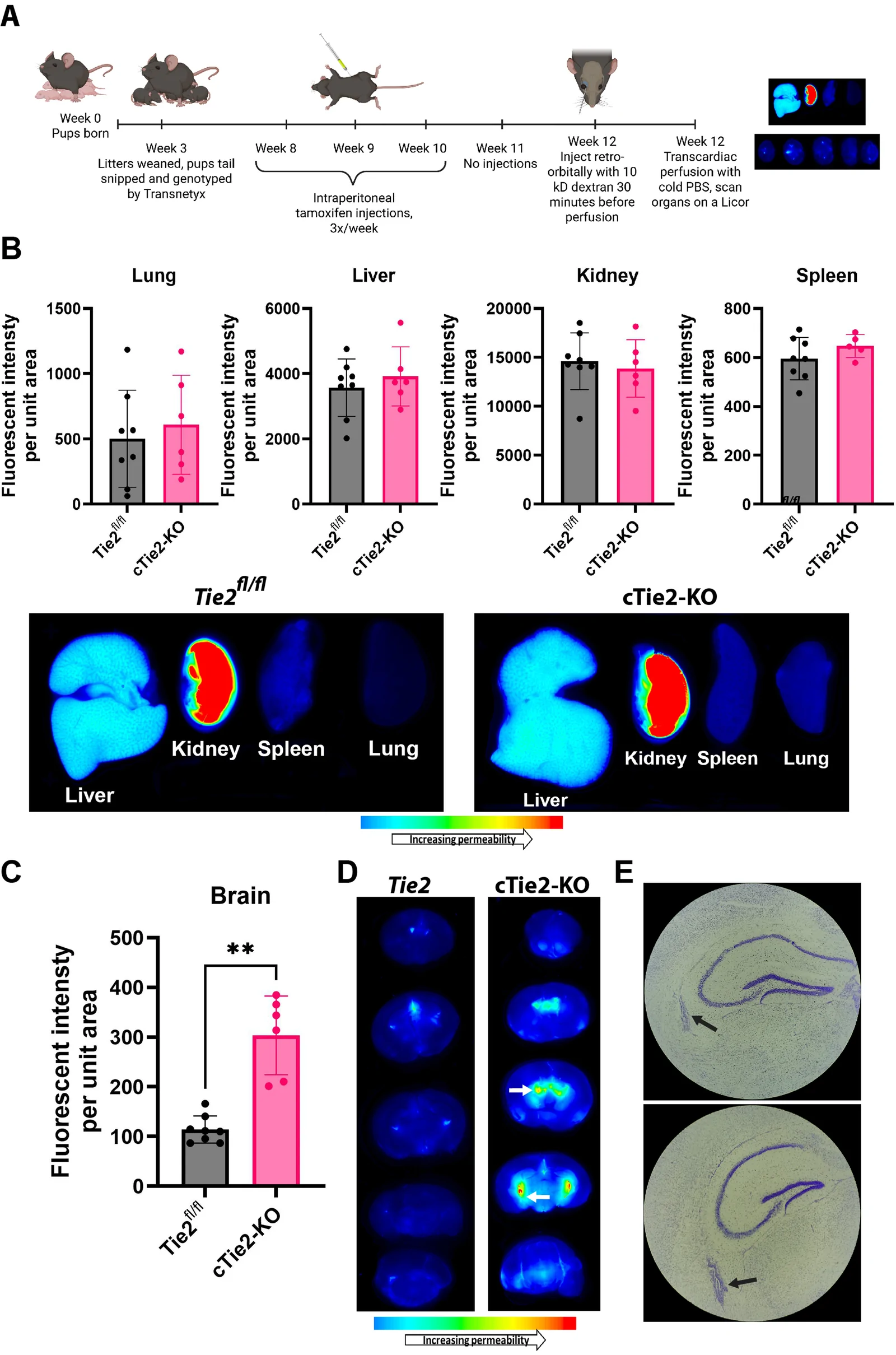
Loss of Tie2 increases vascular permeability in the brain without affecting the peripheral organ permeability in cTie2-KO mice. (**A**) Diagram of the procedure of the permeability assay. (**B**) Graphs of the analysis of the peripheral organs scanned show no changes in fluorescent intensity per unit area. Unpaired two-tailed t-test, columns show individual values and mean ± SD, *n* = 8 *Tie2*^*fl/fl*^, *n* = 6 cTie2-KO. Representative images of scans of *Tie2*^*fl/fl*^ and cTie2-KO organs show no increase in dye intensity in knockout mice as compared to *Tie2*^*fl/fl*^ littermates. (**C**) Brain slices scanned showed a significant increase in fluorescent intensity per unit area in cTie2-KO mice. Unpaired two-tailed t-test, columns show individual values and mean ± SD, *n* = 8 Tie2^fl/fl^, *n* = 6 cTie2-KO, **p ≤ 0.01. (**D**) Representative images of the scans show increased dye intensity in the knockout mice, with highest intensity shown in the lateral ventricles (white arrow). (**E**) Cresyl violet staining of brain tissue shows the location of the lateral ventricles (black arrow). *[Diagram created with BioRender.com]*

After confirmation of endothelial specific Tie2 loss, we tested the barrier permeability of the organs to check if this loss impacts baseline vascular function. Organ permeability and blood brain barrier (BBB) integrity were assessed across all organs in cTie2-KO and littermate controls (Fig. 6A). The intensity of dextran dye per tissue area did not change in the peripheral organs between both genotypes (Fig. 6B). While peripheral organs were not affected, we saw significantly increased extravasation of fluorescently tagged dextran in the brains of cTie2-KO mice as compared to *Tie2*^*fl/fl*^ mice (Fig. 6C). Notably, not only was the brain the most affected organ it was also structurally limited to only ventricular leakage (Fig. 6D, E).

## Discussion

In this study, we describe the generation and characterization of an endothelial-specific Tie2 knockout mouse. In the cTie2-KO mice there was a 73–96% reduction of *Tie2* mRNA expression in the brain, lung, liver, kidney, and spleen, which was accompanied by a 49–93% reduction of Tie2 protein in the same organs as compared to the *Tie2*^*fl/fl*^ mice. Loss of Tie2 expression did not affect mRNA levels of the Tie2 associated proteins. Furthermore, immunocytochemical double staining of tissues confirmed a loss of Tie2 expression specific to the endothelium in all organs, including the spleen. Since Tie2 is integrally linked into regulation of vascular integrity and permeability, we sought to evaluate the effect of Tie2 deletion on organ permeability. There was a significantly increased dextran extravasation in the brains of cTie2-KO mice versus their *Tie2*^*fl/fl*^ littermate controls without affecting permeability in peripheral organs examined. Our model overall shows substantial decrease in Tie2 at both gene and protein expression level in cTie2-KO mice, as compared to *Tie2*^*fl/fl*^ littermate controls in all the organs assayed. Furthermore, this inducible deletion was restricted to the endothelium.

Our study focused on the generation of a conditional homozygous endothelial specific Tie2 knockout mouse. Animals without sufficient Tie2 expression during development cannot survive, so subsequent models need to account for that when developing a knockout mouse. Previous studies describing Tie2 KO mouse models have faced challenges in the creation of a complete knockdown model. Dumont et al. reported on a Tie2 knockout mouse deleting the last 52 base pairs of exon 1, creating a heterozygous Tie2 knockout mouse. Any homozygous embryos from their model died in utero, leading to the conclusion that Tie2 is a crucial part of vascular development [2]. Two studies reported on a Tie2 knockout mouse model, the first focused on Tie2 silencing in the pericytes using *Ng2* as a Cre promoter [11], and the second focused on aortic endothelial cells using *Bmx* as a Cre promoter [12]. Both of these studies focused on the germline or inducible deletion of *Tie2* exon 1. The former study did not induce knockout in the endothelium. While the latter used an inducible Tie2 deletion, it took a narrower view focused on only the arterial endothelium in atherosclerotic diseases. Having an inducible knockout model has an advantage that it permits the development of a normal vasculature before perturbing the expression of Tie2. This allows us to circumvent the embryonic lethality associated with homozygous germline Tie2 knockout. Previously, using the *end-SCL-cre-ER*^*T*^ mouse model to delete exon 9 of *Tie2*, we have shown heterogeneous knockdown across multiple organs but this was specific to hematopoietic stem cells [13] as opposed to the multiorgan focus of a systemic mature endothelial knockout in the current study.

The focus of the current study was to create a multi-organ endothelial knockout of Tie2. To this end we crossed *Cdh5-creERT2*^*(+/−)*^ mice with *Tie2*^*fl/fl*^ mice. Cre-ERT2 expression was controlled by the vascular endothelial cadherin (Cdh5) promoter, which has previously been shown to demonstrate high levels of Cre recombinase gene deletion when induced by tamoxifen [14]. We sought to create an inducible homozygous cTie2-KO mouse to get high levels of Tie2 knockout in all microvascular endothelial cells throughout the organs without encountering the embryonic lethality as created by germline knockout. Tamoxifen has not been shown to have a long term deleterious effect on the endothelium, and the corn oil used to administer it was selected as it is minimally inflammatory when injected intraperitoneally as opposed to corn or mineral oils [15, 16]. Additionally, having its expression controlled by the Cdh5 promoter restricts the knockout to only the mature endothelial cells.

The Tie2 receptor is expressed primarily in the endothelium, with smaller amounts expressed in other cell types. In the brain, Tie2 is expressed in the microglia, a small population of pericytes and some subsets of neurons, such as Purkinje cells [11, 17, 18]. In the lung Tie2 is primarily expressed in the vascular endothelium and pericytes [11], and is upregulated during times of severe inflammation where it can also be expressed in other cell types, such as airway epithelial cells and Tie2 expressing macrophages (TEMs) [19]. TEMs can also be found in the liver and spleen [19–21]. Tie2 is in liver sinusoidal endothelial cells, where it is involved in regulating material exchange [22–24]. Similarly, in the kidney vasculature Tie2 is in the glomerular endothelial cells and podocytes, also involved in maintaining the kidney’s barrier and filtration functions [25, 26]. The most critical period of Tie2 signaling is during development, where it contributes to blood vessel remodeling and the tightening and stabilization of mature blood vessels [1, 27]. In later periods, Tie2 upregulation and activity is critical to neovascularization such as ovulation and wound healing [28, 29]. We have previously shown an inducible Tie2 knockout mouse model using the *end-SCL-CRE-ERT* mouse for endothelial specific knockdown. In that mouse we saw a 55% decrease in Tie2 mRNA and 65% protein reduction in the lung, and a 25% Tie2 mRNA reduction in the kidney with no protein reduction observed in the whole tissue. Comparing this to the current Cdh5-creERT2 model, we consistently observed a significant vascular specific Tie2 protein reduction not only in the kidney and lung as examined in our previous study, but also in the brain, spleen and liver [13].

In this study, *Tie2* mRNA was significantly decreased in the vascular beds of all organs assayed, with a 95% (mean *Tie*^*fl/fl*^ = 0.81; mean cTie2-KO = 0.04) decrease in the brain, 96% in the liver (mean *Tie*^*fl/fl*^ = 0.59; mean cTie2-KO = 0.02), 73% in the lung (mean *Tie*^*fl/fl*^ = 0.31; mean cTie2-KO = 0.08), 92% in the kidney (mean *Tie*^*fl/fl*^ = 0.70; mean cTie2-KO = 0.06) and 84% in the spleen (mean *Tie*^*fl/fl*^ = 0.0002; mean cTie2-KO = 3.03e^− 5^). This was accompanied by a significant decrease in Tie2 protein expression, with 93% (mean *Tie*^*fl/fl*^ = 0.12; mean cTie2-KO = 0.01) reduction in the brain, 91% in the liver (mean *Tie*^*fl/fl*^ = 0.50; mean cTie2-KO = 0.04), 73% in the lung (mean *Tie*^*fl/fl*^ = 9.09; mean cTie2-KO = 2.42), 84% in the kidney (mean *Tie*^*fl/fl*^ = 1.45; mean cTie2-KO = 0.23) and 49% in the spleen (mean *Tie*^*fl/fl*^ = 51; mean cTie2-KO = 0.26). Notably while we saw an 84% reduction of *Tie2* mRNA expression in the spleen, there was a much lower decrease in Tie2 protein in the spleen as compared to the other organs assayed. This discrepancy could be explained by the heterogeneity of cell types in the spleen, such as hematopoietic stem cells, pericytes, and monocytes [11, 30, 31] for which we were unable to correct due to the significantly reduced levels of *Pecam-1* mRNA in cTie2-KO mice. Furthermore, we observed differential amounts of Tie2 expression at mRNA and protein levels across multiple organs. The maximal mRNA expression in *Tie2*^*fl/fl*^ was observed in the brain, however there were extremely low levels of Tie2 protein in the brain as compared to the lungs. Interestingly, significantly higher levels of Tie2 protein were observed in the lungs and kidneys as compared to the other organs.

In quiescent conditions, the phosphorylation of Tie2 as facilitated by Ang1 contributes to stability of the mature vasculature [28, 29]. Ang2 competitively binds to the Tie2 receptor and blocks the binding of Ang1, destabilizing the vasculature and playing an important role in angiogenesis and vascular remodeling [32]. Ang2 blockers and Tie2 agonists have been examined for preventing tumor angiogenesis and cancer cell metastasis [6, 33]. Additionally, tumor cells have been found to also be a source of Ang2 that supports metastasis [34]. Ang1 supplementation to stabilize the vasculature has been proposed as a supplement to chemotherapeutics to prevent metastasis of tumors, and to stabilize the host vasculature [35]. In infectious disease models, reduction of Tie2 expression across multiple infection types leads to weakened endothelial barrier function and reduced efficacy of Ang1 signaling. The loss of the downstream effects of Tie2 signaling, including Akt activation, creates alterations in signaling leading to barrier weakening [36]. In *Tie2*^*fl/fl*^ mice, we observed higher levels of Ang1 mRNA in the brain as compared to the other tissues we assayed. The BBB is an incredibly tight barrier as compared to the peripheral organs. Hence, enriched signaling of Ang1 would help to stabilize and maintain the tight junctions of the brain endothelium [9]. In contrast, we saw the highest level of Ang2 in the kidney. The kidney is a highly permeable organ, as it is heavily involved in filtration of the blood and removal of waste products. As such, a highly active Tie2 signaling pathway could be counterproductive for normal kidney function.

In the peripheral organs loss of Tie2 had no impact on dextran extravasation. Earlier work showed that inflammatory stress in vivo leads to a temporary loss of Tie2 in the kidneys, but did not impact barrier function in the glomeruli [37]. In a model of acute stress, loss of Tie2 did not materially impact the barrier of the kidneys despite a marked loss in the protein level. As these data and other models show acute loss of Tie2 in the peripheral organs does not have an immediate effect on vascular leakage [38], it may be interesting to investigate the effect of Tie2 deletion in a more prolonged state of disease, or a later stage of disease in the peripheral organs. Crucially, in the brain, we saw extravasation of dextran into the parenchyma, specifically in the region of the lateral ventricles. The ventricles of the brain are lined with ependymal cells, which interact with peri-ependymal blood vessels and are reactive to Ang1 signaling [39, 40]. Tanycytes are specialized ependymal cells with processes that directly contact blood vessels found lining the third and fourth ventricles. Ependymal cells secrete Ang1 directly. Tanycytes in particular have direct contact and signaling with the brain capillaries [40]. The ependymal signaling in these ventricles behaves in a different role than in the vascular beds of other organs, as its loss affected the permeability of the ventricles differently than other tissues. While Tie2 is shown to be reduced in all parts of the brain the effect on blood brain barrier permeability is interestingly limited to only the lateral ventricles in the brain, indicating a variability in its function not only between tissues but within the vasculature of the brain itself.

Existing studies have shown the importance of the Tie2 pathway in the brain, and further research is needed to fully characterize how the Tie2 pathway functionally impacts the brain endothelium during TBI and other brain injuries. Recently, the Tie2 pathway has been implicated in TBI as a potential target for therapeutic intervention and understanding how the Tie2 pathway contributes to BBB stability [41]. Age-related differential responses to Tie2 signaling were observed after TBI. In juvenile mice, blocking Tie2 signaling after TBI worsened injury severity and BBB stability [41]. Furthermore, administration of Ang1 in rats after subarachnoid hemorrhage increases BBB stability and improves neurological outcomes [42]. We have recently shown that Tie2 activating antibodies in vitro decrease thrombin induced brain endothelial cell permeability. In an in vivo murine model of TBI, the decoy peptide Tie2-Fc increases BBB permeability after TBI. Thus highlighting that Tie2 is involved in regulating vascular permeability in the BBB [43]. Integrating cTie2-KO mouse into this model will help us further understand the role of Tie2 in BBB maintenance in health, trauma, and disease. This mouse model holds promise for the ability to investigate Tie2 in the context of various vascular disease conditions, including central nervous system vasculature, neurodegenerative diseases, Alzheimer’s, dementia, Parkinson’s and macular degeneration. A significant advantage of this mouse is the ability to examine Tie2 in the vasculature of multiple organs in health and disease.

## Conclusions

We generated an endothelium specific Tie2 conditional knockout mouse, that displayed significant deletion of Tie2 at mRNA and protein levels across multiple organs. This animal model enables us to investigate the role of Tie2 in diseases characterized by vascular dysfunction as well as trauma, such as TBI.

## Electronic supplementary material

Below is the link to the electronic supplementary material.

Supplementary Material 1: Supplemental Fig. 1: *Pecam-1* mRNA normalized to *Gapdh* mRNA to confirm its use as a viable way to normalize other markers to *Pecam-1*. *Pecam-1* was normalized to *Gapdh* and then results were analyzed using unpaired two-tailed t-tests. (A-D) *Pecam-1* showed no change in expression between *Tie2*^*fl/fl*^ and cTie2-KO mice in the brain, lung, liver, and kidney. (E) *Pecam-1* showed significant reduction in expression in the spleen. Unpaired two-tailed t-tests, *n* = 4–5 mice per group, *p ≤ 0.05

Supplementary Material 2: Supplemental Fig. 2: Visualization of individual stains of Pecam-1 and Tie2 by immunocytochemistry. *Tie2*^*fl/fl*^ sections show Tie2 expression (red) and Pecam-1, a pan-endothelial marker (green). Individual images of both stains show a consistent presence of Pecam-1, but a loss of Tie2 signal in (A) Brain cortex (B) Lung (C) Liver (D) Kidney (E) Spleen. Scale bar shown in bottom right image.

## Data Availability

Data available upon request.
